# Cardiovascular disease surveillance using electronic medical records: a scoping study

**DOI:** 10.23889/ijpds.v9i5.1677

**Published:** 2024-09-18

**Authors:** Kiarash Riazi, Adam Virani, Eshnaa Aujla, Jie Pan, Seungwon Lee, Elliot A. Martin, Hude Quan, Na Li

**Affiliations:** 1 Department of Community Health Sciences, Cumming School of Medicine, University of Calgary; 2 Centre for Health Informatics, Cumming School of Medicine, University of Calgary; 3 Libin Cardiovascular Institute, University of Calgary; 4 Provincial Research Data Services, Alberta Health Services

## Objective

Cardiovascular diseases (CVD) are the leading cause of mortality and morbidity worldwide. Traditionally, disease surveillance relies on data from surveys, registries, and administrative databases. As medical records undergo global digitization, electronic medical records (EMRs) are emerging as a crucial reservoir of real-world data. However, the extent EMRs are used in CVD surveillance is unknown. We are conducting a scoping review to assess the current state and effectiveness of EMR-based CVD surveillance worldwide.

## Approach

Following the guidelines of the Preferred Reporting Items for Systematic Reviews and Meta-analyses extension for Scoping Reviews, we searched MEDLINE and EMBASE bibliographic databases to capture studies on prevalence, incidence, and trend measurements of CVDs using EMRs. Assessed factors include CVD types, modelling methodologies, data linkages, advantages, disadvantages, challenges, and solutions. Due to the qualitative nature of the review, collected data will be narratively synthesized to present overall perspectives.

## Results

Our search algorithm yielded 11,979 citations, of which 5,886 abstracts were selected for screening, in progress at the time of this submission. The interim results indicate that most eligible reports came from the USA (49%), followed by the UK (13%), China (6%), Spain (4%) and Canada (3%). The most common diseases were coronary artery diseases (29%), followed by hypertension (26%), stroke (21%), and heart failure (15%).

## Conclusions

Surveillance of CVD is crucial for prevention and health policy development. While EMRs can be a data source for surveillance, such potential has yet to be fully realized.

## Implications

This study will inform existing research challenges and future opportunities of EMR-based CVD surveillance.

